# Acupuncture vs. Pharmacological Prophylaxis of Migraine: A Systematic Review of Randomized Controlled Trials

**DOI:** 10.3389/fneur.2020.576272

**Published:** 2020-12-15

**Authors:** Carlo Maria Giovanardi, Michela Cinquini, Marco Aguggia, Gianni Allais, Manuela Campesato, Sabina Cevoli, Fabio Gentili, Annunzio Matrà, Silvia Minozzi

**Affiliations:** ^1^Italian Federation of Acupuncture Societies, Bologna, Italy; ^2^Laboratory of Clinical Research Methodology, Istituto di Ricerche Farmacologiche Mario Negri IRCCS, Milan, Italy; ^3^SOC Neurologia e Centro Cefalee, Ospedale Cardinal Massaia, Asti, Italy; ^4^Department of Surgical Sciences, Women's Headache Center, University of Turin, Turin, Italy; ^5^UO Anaestesia and Pain Therapy Unit Melotti, Department of Emergency and Urgency, Bologna, Italy; ^6^IRCCS, Istituto delle Scienze Neurologiche di Bologna, Bologna, Italy; ^7^General Practitioner, Specialist in Internal Medicine, Bologna, Italy; ^8^General Practitioner, Bologna, Italy

**Keywords:** acupuncture—therapy, pharmacological prophylaxis, migraine, systematic review, RCTs

## Abstract

**Introduction:** Migraine is a chronic paroxymal neurological disorder characterized by attacks of moderate to severe headache and reversible neurological and systemic symptoms. Treatment of migraine includes acute therapies, that aim to reduce the intensity of pain of each attack, and preventive therapies that should decrease the frequency of headache recurrence. The objective of this systematic review was to assess the efficacy and safety of acupuncture for the prophylaxis of episodic or chronic migraine in adult patients compared to pharmacological treatment.

**Methods:** We included randomized-controlled trials published in western languages that compared any treatment involving needle insertion (with or without manual or electrical stimulation) at acupuncture points, pain points or trigger points, with any pharmacological prophylaxis in adult (≥18 years) with chronic or episodic migraine with or without aura according to the criteria of the International Headache Society.

**Results:** Nine randomized trials were included encompassing 1,484 patients. At the end of intervention we found a small reduction in favor of acupuncture for the number of days with migraine per month: (SMD: −0.37; 95% CI −1.64 to −0.11), and for response rate (RR: 1.46; 95% CI 1.16–1.84). We found a moderate effect in the reduction of pain intensity in favor of acupuncture (SMD: −0.36; 95% CI −0.60 to −0.13), and a large reduction in favor of acupuncture in both the dropout rate due to any reason (RR 0.39; 95% CI 0.18 to 0.84) and the dropout rate due to adverse event (RR 0.26; 95% CI 0.09 to 0.74). Quality of evidence was moderate for all these primary outcomes. Results at longest follow-up confirmed these effects.

**Conclusions:** Based on moderate certainty of evidence, we conclude that acupuncture is mildly more effective and much safer than medication for the prophylaxis of migraine.

## Introduction

Migraine is a chronic paroxymal neurological disease characterized by moderate to severe attacks of headache and reversible systemic and neurological symptoms. The typical state phase includes phonophobia, photophobia, gastrointestinal symptoms such as nausea, and vomit, and cutaneous allodynia (1).

Overall, migraine is a common form of disabling primary headache (1) (ref ICHD-3) and it is among the most prevalent disorder worldwide, affecting about 15% of adults in Western countries. Its prevalence is higher in the age group 35–39 years and among females. About 2.5% of subjects with episodic migraine progress to chronic migraine (CM) every year. CM, the most disabling form of migraine, currently affects up to 2% of the population worldwide.

Migraine is considered among the top five causes of disability by the Global Burden of Disease study with about one billion individuals affected worldwide. The GBD 2016 study reported that migraine is the first cause of years lived with disability (YLDs) both in males and females in the age group 15–49 years (2), afflicting people in the active period of their life, leading to a reduction of quality of life and relevant disability, with significant social consequences.

Migraine also leads to important use of health care resources, high direct and indirect costs, and work loss, with a significant societal impact.

A recent review on the burden of this disease highlighted that the total cost of migraine in USA was more than 2,500$ per year, and that the cost of CM was more than 8,200$ per year: according to this study, 60–64% of its costs were attributable to direct medical costs. In a recent European study the average direct cost of EM was estimated as 746€ per year, while those of CM were estimated as 2,427€ per year (3).

Treatment of migraine includes both therapies of the acute attacks, with the aim to reduce the intensity of pain of each migraineous attack, and prophylactic treatments, with the aim to reduce the frequency of headache recurrence. Considering both the evidence for efficacy and the risk of potential side effects, drugs with the most favorable profiles are amitriptyline, beta-blockers, candesartan, flunarizine, onabotulinumtoxinA (for CM), pizotifene, and topiramate. In addition, calcitonin gene-related peptide (CGRP) receptor monoclonal antibodies were recently approved for migraine prophylaxes (4).

Despite the progresses in drug treatment, patients are often still unsatisfied because of the insufficient control of pain or the disturbing adverse events that limit patients' compliance.

In the last decades, acupuncture has been suggested as a valid non-pharmacological alternative for migraine prophylaxis and its use in Western countries has increased considerably.

### Mechanisms of Action

According to some authors, acupuncture carry out its analgesic effects by interacting with the hypothalamic-pituitary-adrenal axis and the endogenous opioid system, known to be important mediators of the stress response to pain (5). Several studies demonstrated that acupuncture activate the release of opioid in the central nervous system (6–10). Gao et al. (6) report that “*Release of these peptides corresponds to long-lasting activation of ascending sensory tracks, thereby relieving an array of pain conditions*.” Furthermore, using a migraine rat model, researchers observed that the expression of CGRP in the trigeminal ganglion, which is considered a migraine trigger factor (11), could be depressed by the electro-acupuncture treatment (12). In addition, acupuncture was demonstrated to restore the descending pain modulatory system, impaired in patients affected by migraine, by decreasing the resting state of functional connectivity between the rostral anterior cingulate cortex/medial prefrontal cortex and the periaqueductal gray, which is correlated with the intensity of pain during the attacks (13).

To understand the mechanisms of action of acupuncture, as well as its local, remote, and long-term effects, a key may be the reaction to needles insertion, called “de qi” and widely judged as an important factor affecting the therapeutic effect of acupuncture. “De qi” includes both a typical needling sensation, sensed by the patient, and a “needle grasp” noticed by the provider: the acupuncturist perceives tearing and augmented resistance to additional movement of the needle. Langevin hypothesized that “*needle grasp is due to mechanical coupling between the needle and connective tissue with winding of tissue around the needle during needle rotation and needle manipulation transmits a mechanical signal to connective tissue cells via mechano-transduction*” (14).

In conclusion, although the mechanism of action of acupuncture doesn't have yet a definite explanation, acupuncture has a scientific basis built on a series of studies that have been conducted over the last decades: there are neurobiological models that could elucidate how acupuncture obtain its therapeutic effect in several clinical settings.

The efficacy of acupuncture for migraine has been confirmed by several clinical trials and a series of systematic reviews, including a Cochrane Review (15, 16). Consequently, acupuncture is becoming a more accepted form of integrative medicine in the Western countries for the prevention and treatment of migraine (17) and is administered for migraine prophylaxis and chronic pain treatment all over the world (15, 18): migraine and other headaches were the primary indications for acupuncture treatment in 9.9% of US patients (17).

In 2009, a Cochrane review concluded that “*there is consistent evidence that acupuncture provides additional benefit to treatment of acute migraine attacks only or to routine care. Available studies suggested that acupuncture is at least as effective as, or possibly more effective than, prophylactic drug treatment, and has fewer adverse effects*” (15); after 7 years, the update of the same Cochrane review suggested the prevalence of headaches is reduced by combining acupuncture with usual care; it also suggested that there is an effect over sham and that acupuncture may be at least as effective as treatment with prophylactic drugs and it is associated with fewer adverse effects (16).

Since 2016, several new studies have appeared to support the efficacy of acupuncture in migraine prevention (16); acupuncture can be recognized as a valid treatment in Western countries if its specific effects are clearly understood.

The objective of this systematic review was to assess the efficacy and safety of acupuncture for the prophylaxis of episodic or chronic migraine in adult patients when compared to pharmacological treatment.

## Materials and Methods

We performed this systematic review according to the Preferred Reporting Items for Systematic Reviews and Meta-Analyses (PRISMA) statement (19).

### Inclusion Criteria

We included randomized-controlled trials published in western languages that compared any treatment involving needle insertion (with or without manual or electrical stimulation) at acupuncture points, pain points or trigger points, described as acupuncture with any pharmacological prophylaxis in adult (≥18 years) participants with chronic and episodic migraine with or without aura according to the criteria of the International Headache Society (1). Studies on patients with cluster headache or tension-type headache were excluded. We also excluded studies that evaluated acupuncture at specific “microsystems” (e.g., scalp or ear acupuncture), although we included trials using micro-system points in addition to body acupuncture; evaluated other methods of stimulating acupuncture points without needle insertion, for example, acupressure, laser stimulation, or transcutaneous electrical stimulation; injected fluids at acupuncture or trigger points.

Primary outcomes were:

- number of days with migraine per month
- response rate (≥50% frequency reduction documented in a headache diary)
- pain intensity as documented in a headache diary
- dropout from treatment for any reason
- drop out due to adverse event.

Secondary outcomes assessed were:

- quality of life as measured by validated scales (e.g., SF-36; SF-12) (20, 21)
- frequency of migraine attack per month
- disability due to migraine as measured by validated scale (e.g., MIDAS score; Pain disability index) (22, 23)
- use of rescue medication
- number of subjects with at least one adverse event AE.

### Identification of Eligible Trials

Cochrane Database of Systematic Reviews (CENTRAL), Embase, MEDLINE, and ClinicalTrial.gov were searched for eligible studies. Literature search was performed using free text and Mesh terms from inception up to 14 May 2020 without language restriction. The detailed search strategy is reported in the Supplementary Material 1.

### Data Collection and Analyses

Two authors independently screened articles retrieved via the search strategy from the title and abstracts. Potentially relevant studies were acquired in full text and assessed for final inclusion independently by two authors. Any disagreement was discussed with a third author. Two review authors independently extracted data from the studies We extracted the following information: number and characteristics of participants: mean age, % female, duration of disease in years, chronic vs. episodic migraine; details of acupuncture treatments: number of sessions, number of acupoints, achievement of de-chi (an irradiating feeling considered to indicate effective needling), duration of treatment in weeks; type of drugs received by participant of the control group, length of follow-up after the end of treatment, types of outcomes assessed, country where the study was conducted.

Two authors independently assessed risk of bias according to the criteria set out in the Cochrane Handbook for Systematic Reviews of Interventions (24). The following criteria were considered: sequence generation and allocation concealment (selection bias), blinding of participants and providers (performance bias), blinding of outcome assessors (detection bias), incomplete outcome data (attrition bias), and selective outcome reporting (reporting bias). Disagreement between reviewers was resolved by discussion.

We analyzed dichotomous outcomes by calculating the risk ratio (RR) for each trial with the uncertainty in each result being expressed with a 95% confidence interval (CI). We analyzed continuous outcomes by calculating the mean difference (MD) with 95% CI when the studies used the same instrument for assessing the outcome. We used the standardized mean difference (SMD) when the studies used different instruments. We interpreted SMD values with the classification proposed by Cohen et al. (25) where an effect size of 0.2 means a small effect, 0.5 means a medium effect, 0.8 means a large effect.

As we supposed a certain degree of heterogeneity among studies, due to treatment schedules, way in assessing response criteria, risk of bias and other factors which may have affected direction and magnitude of treatment effect, we pooled data used the random effect model for each outcome.

Seeking statistical heterogeneity among studies, the Cochrane *Q*-test was performed, with a significant threshold of alpha = 0.1 and inconsistency among studies was quantified by the I-squared statistic (24); an I square >70% was judged a significant heterogeneity.

Results are depicted in all figures as conventional meta-analysis forest plots. RevMan 5.3 was used for producing forest plot figures (26).

We planned to use visual inspection of funnel plots (plots of the effect estimate from each study against the sample size or effect standard error) to indicate possible publication bias if there were at least 10 studies included in the meta-analysis.

### Subgroup Analysis

Although the STRICTA (Standards for Reporting Interventions in Controlled Trials of Acupuncture) recommendations describe the components of acupuncture procedures (27) better outcomes appear to be associated with a greater numbers of needles and treatment sessions (28) and on the other hand, an insufficient dose of acupuncture may be an obstacle to good patient care (29).

When starting this study, the vast heterogeneity among the contributions available in the literature has soon become evident. The extremely different ways of administering acupuncture made it almost impossible to compare the outcomes of the trials. Consequently, we have decided to establish standard criteria for comparison of data. By introducing the concept of adequate dose of acupuncture already expressed by other authors (30), we have considered the following three parameters:

- number of points needled during each treatment
- de qi response
- number of treatment sessions.

The “de qi” response, that is to say the sensation from needling experienced by the patient, may be reported as numbness (A-beta fiber activation) or as aching, dull, heavy, and warm sensation (A-delta or C fiber activation) (31).

The concept of dose-intensity has thus been introduced and used to group the studies according to the intensity of acupuncture based on the following criteria:

- number of sessions (≥8 vs. <8)
- number of acupoints treated (≥10 vs. <10)
- achievement of de-qi (yes vs. no/not reported).

Acupuncture was judged as at low intensity of only one criterion was met; on medium intensity if two criteria were met; high intensity if all the three criteria were met. Subgroup analyses was performed for intensity of acupuncture.

### Grading of Evidence

We assessed the overall quality of the evidence for the primary outcomes using the five GRADE domains (study limitations, consistency of effect, imprecision, indirectness, and publication bias) according to the GRADE approach (32).

Based on the above domains, the GRADE system uses the following criteria to grade the evidence:

High: we are very confident that the true effect lies close to that of the estimate of the effect.

Moderate: we are moderately confident in the effect estimate: the true effect is likely to be close to the estimate of the effect, but there is a possibility that it is substantially different.

Low: our confidence in the effect estimate is limited: the true effect may be substantially different from the estimate of the effect.

Very low: we have very little confidence in the effect estimate: the true effect is likely to be substantially different from the estimate of effect.

The existing evidence was summarized in a “Summary of Findings” table that provides key information about the magnitudes of relative and absolute effects of the interventions, the amount of available evidence and the certainty of available evidence (33).

## Results

**Figure 1:**
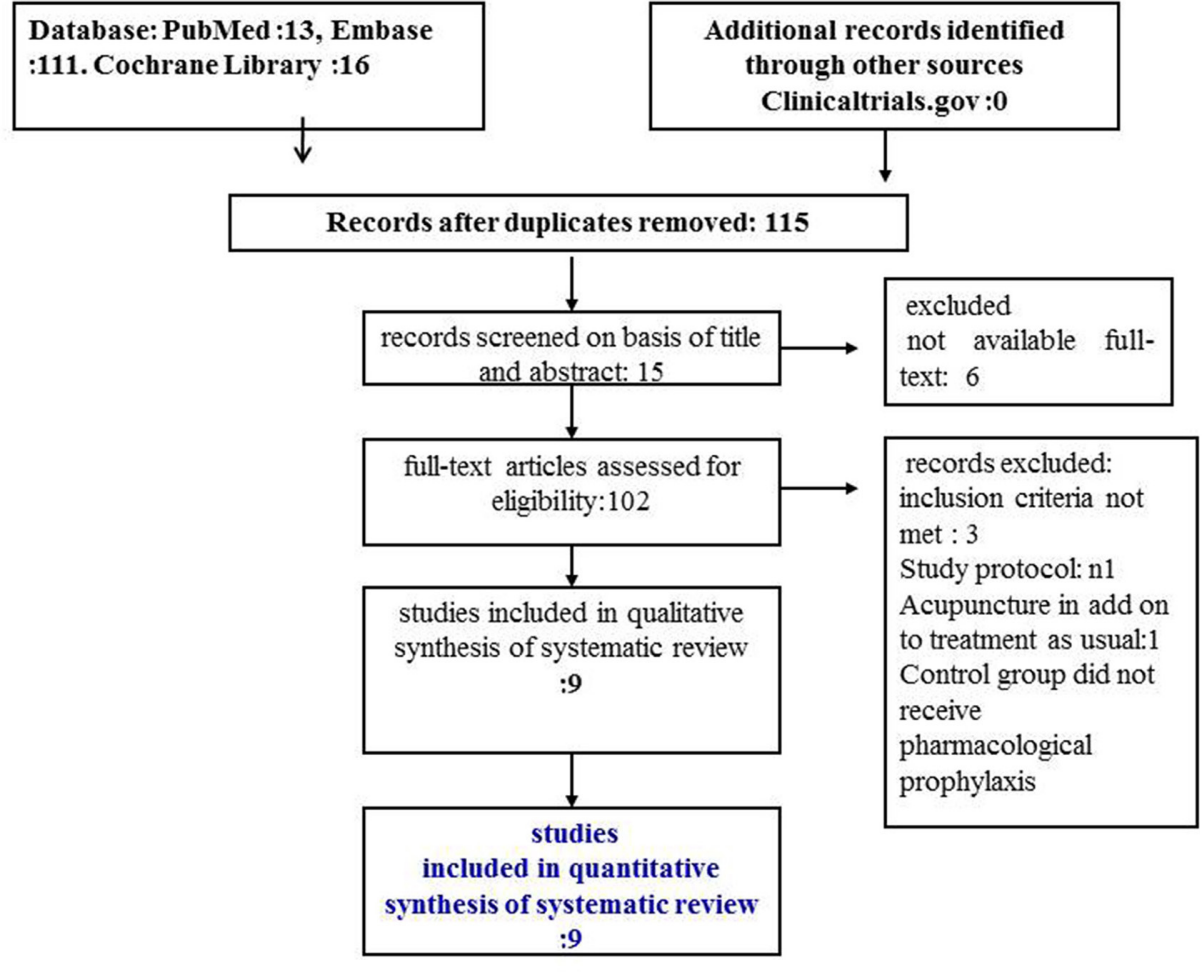
PRISMA flow diagram.

The database searches retrieved 115 records after duplicate were removed. Eighteen studies were judged as potentially relevant. For 6 records we were unable to retrieve the full text. Three articles were excluded because they did not meet the inclusion criteria (34–36). Nine randomized trials were finally included (31, 37–44) (Figure 1). These trials included 1,484 patients. Participants were recruited from outpatient departments in 6 studies (31, 38, 39, 41–43); they were partly respondents to a newspaper advertisement, partly referred from general practitioners in one study (40); methods of recruitment was not described in two studies (37, 44).

The mean age of participants was 39.5 year (range 36–47 years). Most participants were female (mean: 81% range: 81–100%). Mean duration of migraine was 13.9 (SD 6.12) years and the mean number of migraine days per month at baseline was 13.3 (SD 7.4), data available only from 5 studies.

The mean duration of acupuncture was 11 weeks (range 4–24 weeks). Acupuncture was judged of high intensity in 6 studies (32, 37–39, 41, 43), of medium intensity in one study (42), of low intensity in 2 studies (40, 44).

Drugs received by the control group were: flunarizine: 3 studies (37, 42, 44); valproate 2 studies (31, 39); metoprolol: 2 studies (40, 41); Topiramate: 1 study (43); either beta blockers, flunarizine or valproate: 1 study (37). One study (31) had a third arm which received botulinum toxin only once in 31 trigger zones over the facial and pericranial muscles, at the total dosage of 155 U. This arm was not considered in our meta-analysis as the total doses and the frequency of injections were judged insufficient to have a therapeutically effect. In fact, the study performed only one botulinum toxin A session while, according the protocol of the PREEMPT (36) and subsequent real-life studies, efficacy was higher after the second and third sessions.

The migraine days per months, the number of attacks per months, pain intensity and use of rescue medications were recorded in all the studies through a headache diary. We reported the results at the end of treatment period, which ranged from 4 to 24 weeks and at the longest available follow up, which ranged from 8 to 26 weeks.

Two studies were conducted in China (42, 44), two in Italy (37, 39) two in Germany (38, 41), one each in Taiwan (43), Iran (31), and Denmark (40). Table 1 summarizes the characteristics and results of each trial.

**Table 1:** Characteristics of included studies.

**References**	**Participants**	**Acupuncture**	**Dose intensity**	**Comparison intervention**	**Treatment duration (weeks)**	**Length of follow up (weeks)**	**Country**	**Funding**
Allais et al. (37)	160 participants with episodic migraine without aura Methods of recruitment: not reported Mean age: 37.8 years (SD 9.8) Female. 100% Mean years of disease: about 20 Mean number of migraine days per month: not reported	*n*:80*n* of session: 12*n* of acupoints treated: 10Achievement of de-qi: yes	High	*n*:80Flunarizine 10 mg/die	24	24	Italy	Not reported
Diener et al. (38)	960 participants with episodic migraine (with and without aura) Methods of recruitment: from outpatient clinics Mean age: 37 years (SD 10.5) Female:83.8% Mean years of disease: 16.3 (SD: 12) Mean number of migraine days per month: not reported	*n*:313(339 assigned to sham group)*n* of session: 12*n* of acupoints treated: 10Achievement of de-qi: yes	High	*n*:308Either beta blockers, flunarizine or valproate, doses not reported	6–13	26	Germany	German public-healthInsurance companies
Facco et al. (39)	100 participants with episodic migraine without aura Methods of recruitment: from outpatient clinics Median years: 38 (IQR: 32–44) female:65.8 % Median years of disease: 4 (IQR:3.5) Mean number of migraine days per month: not reported	*n*:50	High	*n*:50Valproate 600 mg/die	12	24	Italy	Not reported
Hesse et al. (40)	85 participants with episodic migraine (with and without aura) Methods of recruitment: partly respondents to a newspaper advertisement, partly referrals from general practitioners. Mean age: 44.7 years (range 26–70) Female:84.4% Mean years of disease: 23.4 (range 2–55) Mean number of migraine days per month: not reported	*n*:38 (completers; *n*. randomized not reported)	Low	*n*:39 (completers; *n*. randomized not reported)Metoprolol 100 mg/die	17	17	Denmark	Danish Health Foundation and DanishMedical Research Council
Naderinabi et al. (31)	162 participants with chronic migraine (with and without aura) Methods of recruitment: enrolled in Guilan Pain Clinic Mean age: 37.2 years (SD 7.3) Female: 59.3% Mean years of disease: 9.57 (SD: 4.9) Mean number of migraine days per month: 21	*n*:50	High	Arm 1 (*n*:50): valproate 500 mg/dieArm 2 (*n*:50): botulinum toxin, one administration in 31 trigger zones over the facial and pericranial muscles, at the total dosage of 155 U.	8	12	Iran	Research and Technology Vice-Chancellorship of Guilan University of Medical Sciences
Streng et al. (41)	114 participants with episodic migraine (with and without aura) Methods of recruitment: not reported Mean age: 40 years (SD 11.34) Female:88.5% Mean years of disease: 15.7 (SD: 10.34) Mean number of migraine days per month: 5.8	*n*:59	High	*n*:55Metoprolol 100–200 mg/die	12	24	Germany	German social health insurance funds
Wang et al. (42)	140 participants with episodic migraine without aura Methods of recruitment: from outpatient acupuncture departments Mean age: 39.5 years (SD 12) Female:85% Mean years of disease: not reported Mean number of migraine days per month: 6.6	*n*:70	High	*n*:70Flunarizine 10 mg/die in the first 2 weeks and 5 mg/die in the next 2 weeks	4	16	China	Capital Medical Development ResearchFund
Yang et al. (43)	66 participants with chronic migraine (with and without aura) Methods of recruitment: from the outpatient department Mean age: 47.8 years (SD 6.9) Female:89.4 % Mean years of disease: 13.35 (SD 4) Mean number of migraine days per month: 21	*n*:33	Medium	*n*:33Topiramate; start with 25 mg/die, then gradually increase up to maintenance dose of 50 to 100 mg/die	12	12	Taiwan	Hung KuangUniversity and Kuang Tien General Hospital
Zhao et al. (44)	36 participants with chronic migraine (with and without aura) Methods of recruitment: not reported Mean age: 36.1 years (SD 6.25) Female:75% Mean years of disease: 9.85 (SD 3) Mean number of Migraine days per month: 12.15	*n*:18	Low	*n*:18Flunarizine 10 mg/die	4	8	China	Not reported

### Risk of Bias in Included Studies

**Figure 2:**
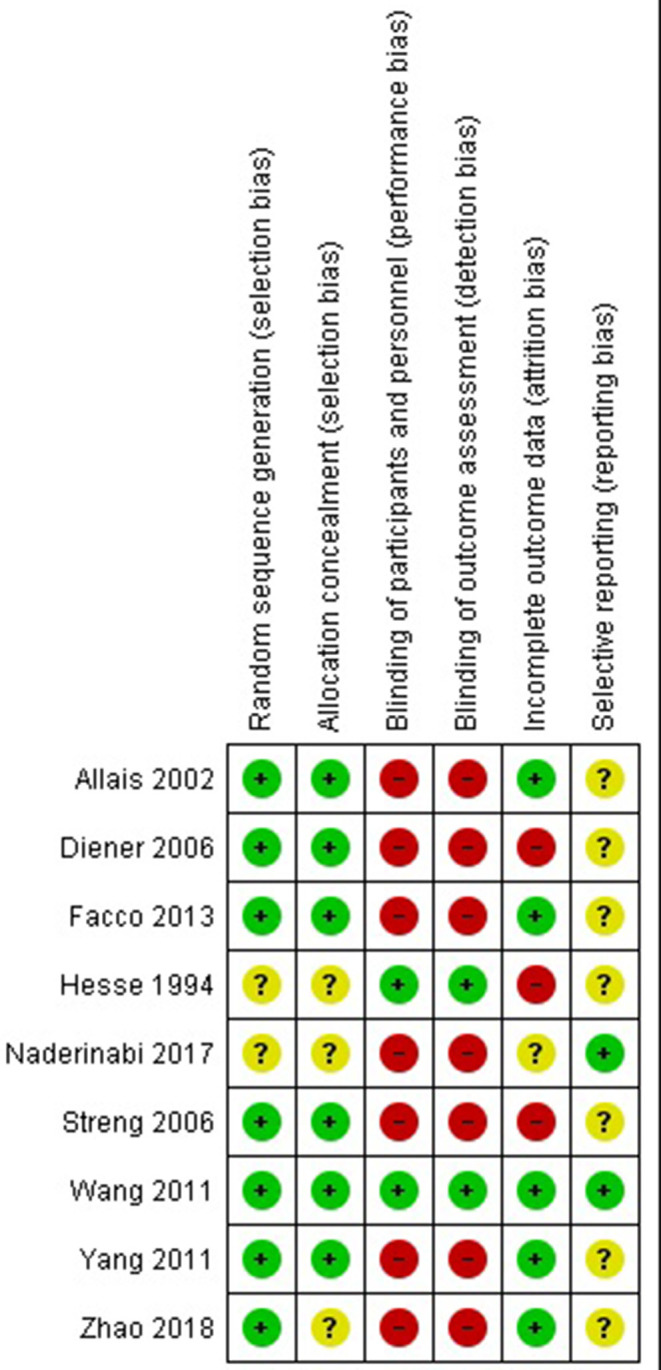
Risk of bias assessment.

Six studies were judged at low risk of selection bias because both the methods for random sequence generation and allocation concealment was appropriate; one study (44) followed ad adequate method for random sequence generation but did not provide information about concealment of allocation. The remaining two studies (31, 40) were judged at unclear risk for selection bias because they did not provide any information about methods followed to generate the random sequence and to conceal the allocation. All but three studies were judged at high risk of both performance and detection bias because they were open label; Two studies (40, 42) used the double-blind double dummy approach and was judged at low risk for both the domains. Three studies were judged at high of attrition bias (38, 40, 41) because of the high number of subjects who dropped out from studies and no attempt to perform an intention to treat analysis. One study (31) did not provide information about subjects dropped out from each group. The study protocol was available only for two studies (31, 42) and the outcomes reported in the final publication coincided with the outcomes listed in the protocol; for all the remaining studies the protocol was not available and they were judged at unclear risk of selective outcome reporting (Figure 2).

### Effects of Interventions

#### Results at the End of Intervention

**Figure 3:**
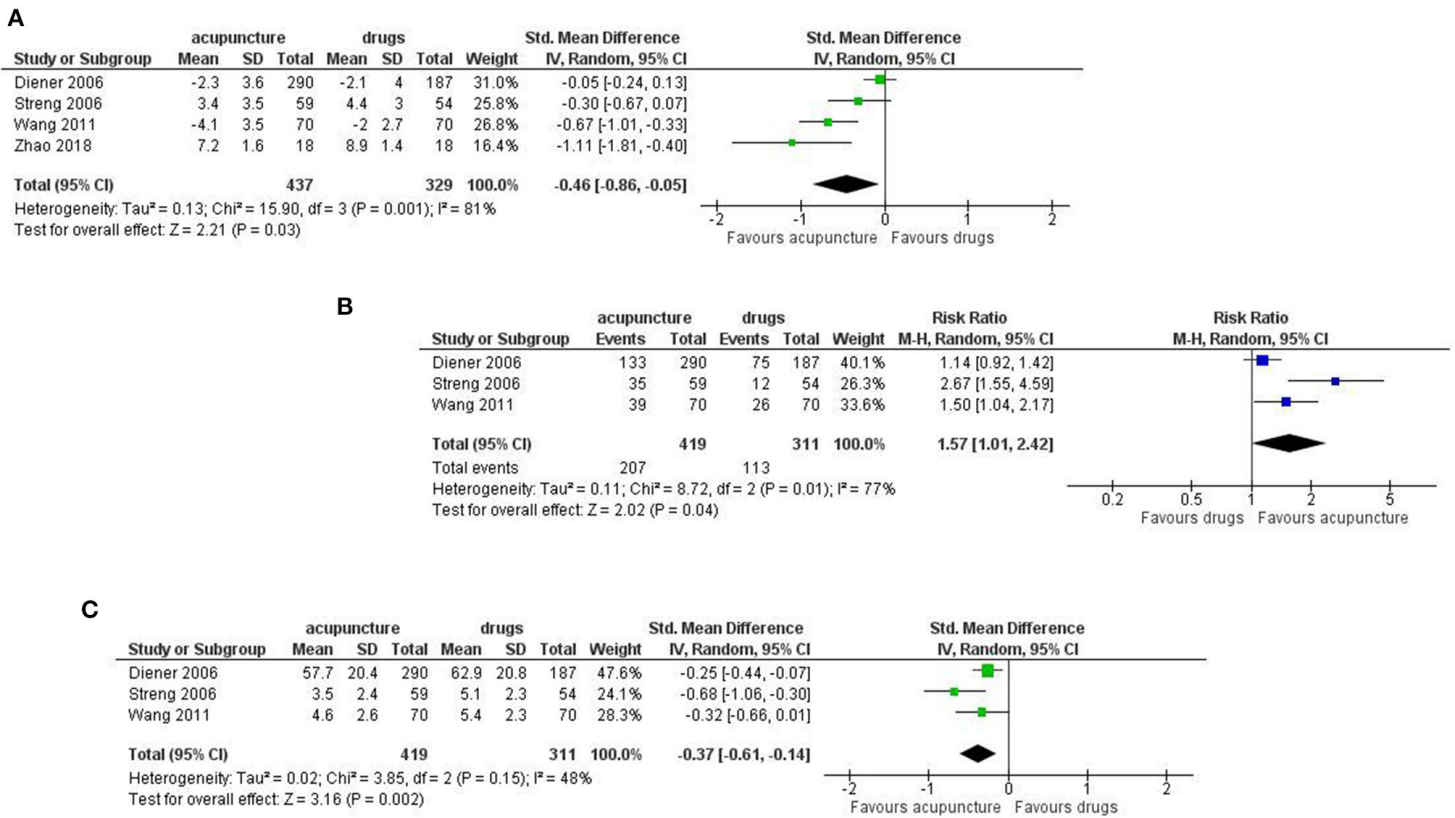
Forest plot of primary outcomes—results at the end of the intervention. **(A)** Number of days with migraine per months; **(B)** Response rate to treatment; **(C)** Pain intensity.

Number of days with migraine per month: we found a small but significant effect in favor of acupuncture (SMD: −0.37; 95% CI −0.64 to −0.11; I^2^ = 71%, 6 studies, 992 participants; Figure 3A).

Response rate: we found a small but significant reduction in favor of acupuncture (RR: 1.46; 95% CI 1.16 to 1.84; I^2^ = 58%, 5 studies, 956 participants; Figure 3B).

Pain intensity: we found a moderate effect in the reduction of pain in favor of acupuncture (SMD: −0.36; 95% CI −0.60 to −0.13; I^2^ = 49%, 3 studies, 730 participants; Figure 3C).

**Figure 4:**
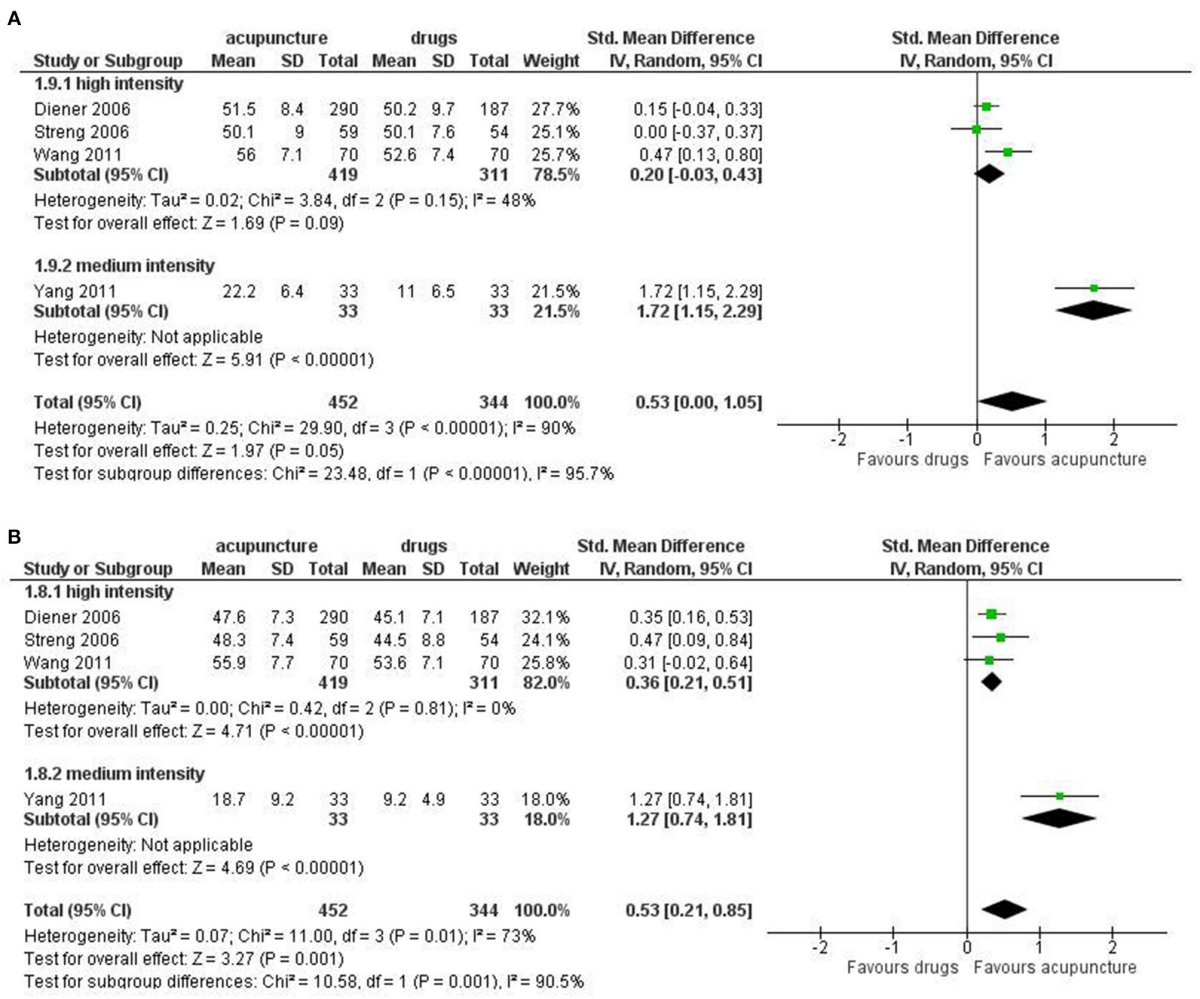
Forest plot of primary outcomes—results at the end of the intervention. **(A)** Dropout due to any events; **(B)** Dropout due to adverse events.

Dropout: we found a strong reduction in favor of acupuncture in both the dropout rate due to any reason (RR 0.39; 95% CI 0.18 to 0.84; I^2^ = 77%, 6 studies, 1,211 participants) and the dropout rate due to adverse event (RR 0.26; 95% CI 0.09 to 0.74; I^2^ = 0%, 6 studies, 646 participants; Figures 4A,B).

Quality of evidence was moderate for all the primary outcomes (Table 2).

**Table 2:** Summary of findings.

**Acupuncture compared to pharmacological prophylaxis for migraine**						
Patient or population: migraine						
Setting: outpatients						
Intervention: acupuncture						
Comparison: pharmacological prophylaxis						
**Outcomes**	Anticipated absolute effects^*^ **(95% CI)**		**Relative effect (95% CI)**	**No of participants (studies)**	**Certainty of the evidence (GRADE)**	**Comments**
	**Risk with pharmacological prophylaxis**	**Risk with acupuncture**				
*N*. days/month follow up: mean 11.5 weeks	–	SMD **0.37 SD lower** (0.64–0.11 lower)^a^	–	992 (6 RCTs) (37, 38, 41–44)	⊕⊕⊕○ MODERATE^b^^,^ ^c^	Acupuncture probably reduce *n*. days/month of migraine
Response rate follow up: mean 13 weeks	40 per 100	**59 per 100** (47–74)	**RR 1.46** (1.16–1.84)	956 (5 RCTs) (37, 38, 41–43)	⊕⊕⊕○ MODERATE^b^^,^ ^d^	Acupuncture probably increase response rate
Pain intensity follow up: mean 9.6 weeks	–	SMD **0.36 SD lower** (0.6–0.13 lower)^e^	–	730 (3 RCTs) (38, 41, 42)	⊕⊕⊕○ MODERATE^b^^,^ ^f^	Acupuncture probably reduce pain intensity
Dropout follow up: mean 12.8 weeks	39 per 100	**15 per 100** (7–33)	**RR 0.39** (0.18–0.84)	1211 (6 RCTs) (37–39, 41–43)	⊕⊕⊕○ MODERATE^b^^,^ ^d^	Acupuncture probably reduce dropout
Dropout due to AEs follow up: mean 12.8 weeks	6 per 100	**2 per 100** (1–4)	**RR 0.26** (0.09–0.74)	646 (6 RCTs) (37, 39–43)	⊕⊕⊕○ MODERATE^g^	Acupuncture probably reduce dropout due to AEs
*N*. days/month at follow-up follow up: range 8–26 weeks	–	SMD **0.46 lower** (0.86–0.05 lower)	–	766 (4 RCTs) (38, 41, 42, 44)	⊕⊕⊕○ MODERATE^h^	Acupuncture probably reduce *n*. days/month with migraine at follow-up
Response rate at follow-up follow up: range 16–26 weeks	36 per 100	**57 per 100** (37–88)	**RR 1.57** (1.01–2.42)	730 (3 RCTs) (38, 41, 42)	⊕⊕⊕○ MODERATE^h^	Acupuncture probably response rate at follow-up
Pain intensity at follow-up follow up: range 16–26 weeks	–	SMD **0.37 lower** (0.61–0.14 lower)	–	730 (3 RCTs) (38, 41, 42)	⊕⊕⊕○ MODERATE ^h^	Acupuncture probably reduce pain intensity at follow-up

* *The risk in the intervention group (and its 95% confidence interval) is based on the assumed risk in the comparison group and the relative effect of the intervention (and its 95% CI)*.

*CI, Confidence interval; SMD, Standardized mean difference; RR, Risk ratio*.

*GRADE Working Group grades of evidence*.

*High certainty: We are very confident that the true effect lies close to that of the estimate of the effect. Moderate certainty: We are moderately confident in the effect estimate: The true effect is likely to be close to the estimate of the effect, but there is a possibility that it is substantially different*.

*Low certainty: Our confidence in the effect estimate is limited: The true effect may be substantially different from the estimate of the effect*.

*Very low certainty: We have very little confidence in the effect estimate: The true effect is likely to be substantially different from the estimate of effect*.

a *In Naderinabi 2017 a statistically significant reduction in days/month of migraine is reported (p = 0.0001)*.

b *High risk of performance and detection bias in all studies but one, high risk of attrition bias in 2 studies*.

c *2 studies (weight = 25%) with short-course pharmacological treatment (4 weeks)*.

d *1 study (weight = 20%) with short-course pharmacological treatment (4 weeks)*.

e *Naderinabi 2017 a statistically significant reduction in pain intensity is reported (p = 0.0001) in the control arm*.

f *1 study (weight = 28%) with short-course pharmacological treatment (4 weeks)*.

g *High risk of performance and detection bias. High risk of attrition bias in 1 study*.

h *High risk of performance and detection bias. High risk of detection bias in 2 studies*.

**Figure 5:**
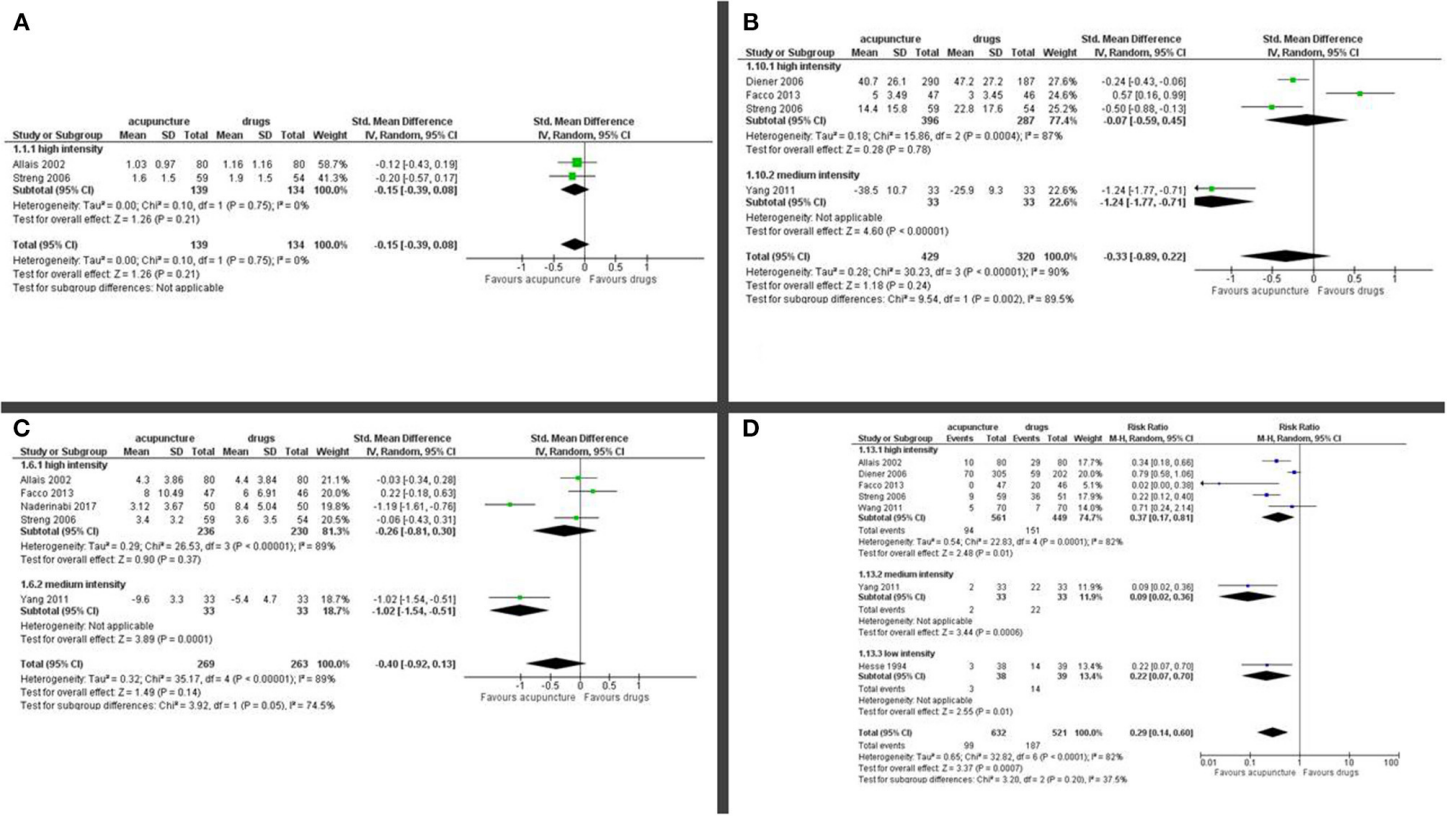
Forest plot of secondary outcomes—results at the end of the intervention. **(A)** Frequency of migraine attack per month; **(B)** Disability; **(C)** Use of rescue medication; **(D)** Number of subjects with at least one adverse event.

For the secondary outcomes we did not find significant difference between treatments in the frequency of migraine attack per month (SMD: −0.15, 95% CI −0.39 to 0.08; I^2^ = 0%, 2 studies, 273 participant); disability (SMD: −0.33, 95% CI −0.89 to 0.22; I^2^ = 90%, 4 studies, 479 participants); use of rescue medication (SMD: −0.40, 95% CI −0.92 to 0.13; I^2^ = 89%, 5 studies, 532 participants); we found a significant difference in favor of acupuncture in the number of subjects with at least one adverse event (RR: 0.29, 95% CI 0.14 to 0.60; I^2^ = 82%, 7 studies, 1,153 participants; Figures 5A–D).

**Figure 6:**
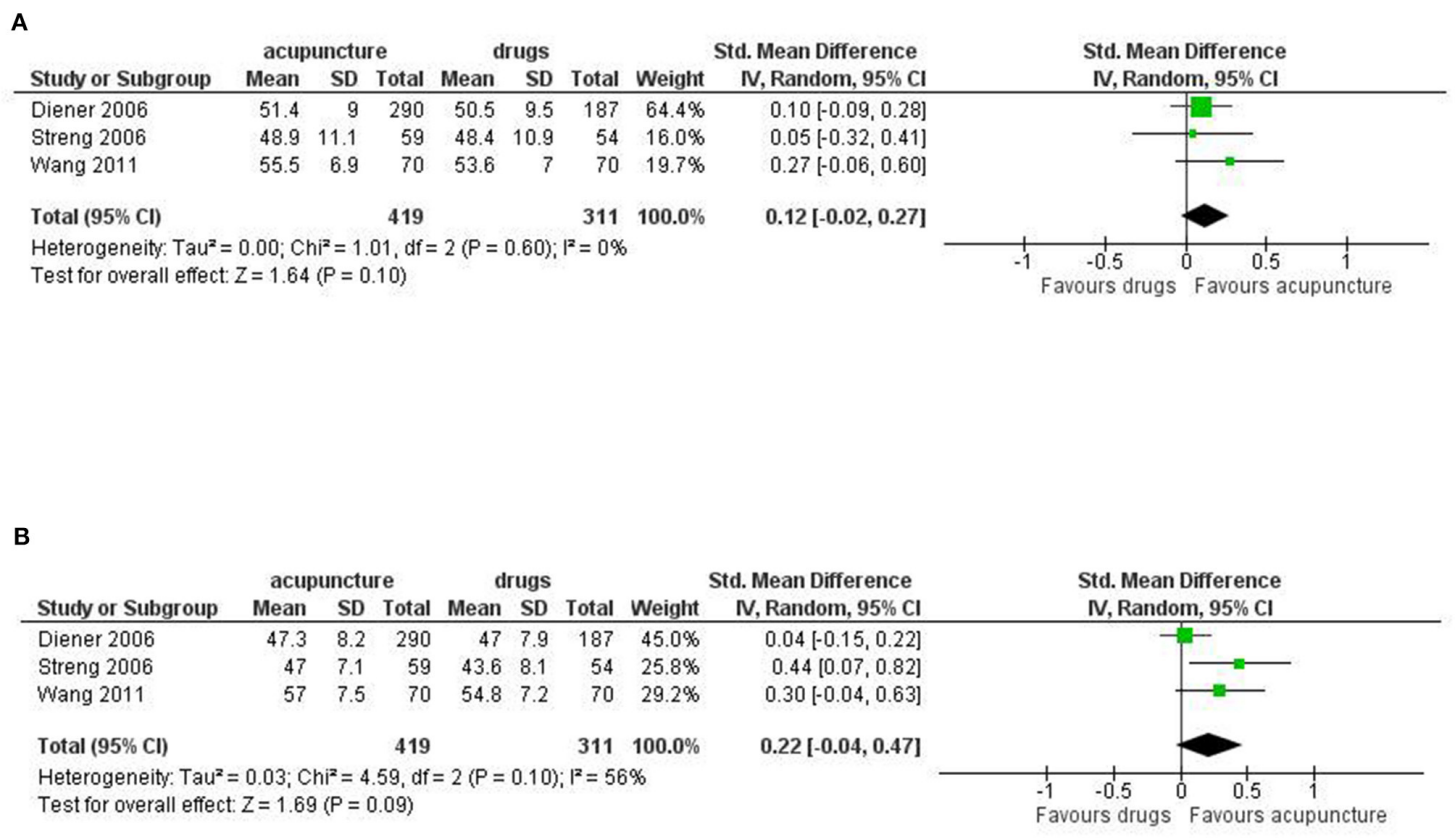
Forest plot of secondary outcomes—results at the end of the intervention. **(A)** Quality of Life: mental health; **(B)** Quality of Life: physical health.

For Quality of life, we found a moderate effect in favor of acupuncture for both the mental health subdomain (SMD: 0.53; 95% CI 0.00 to 1.05; I^2^ = 90%, 4 studies, 796 participants) and physical health subdomains (SMD: 0.53; 95% CI 0.21 to 0.85; I^2^ = 73%, 4 studies, 796 participants; Figures 6A,B).

Quality of evidence was moderate for number of subjects with at least one adverse event and quality of life and low for all the other secondary outcomes.

Subgroup analysis did not reveal important difference in all the primary outcomes between acupuncture of different intensity. However, there were too few studies in the subgroup to allow firm conclusion.

#### Results at Longest Available Follow up

**Figure 7:**
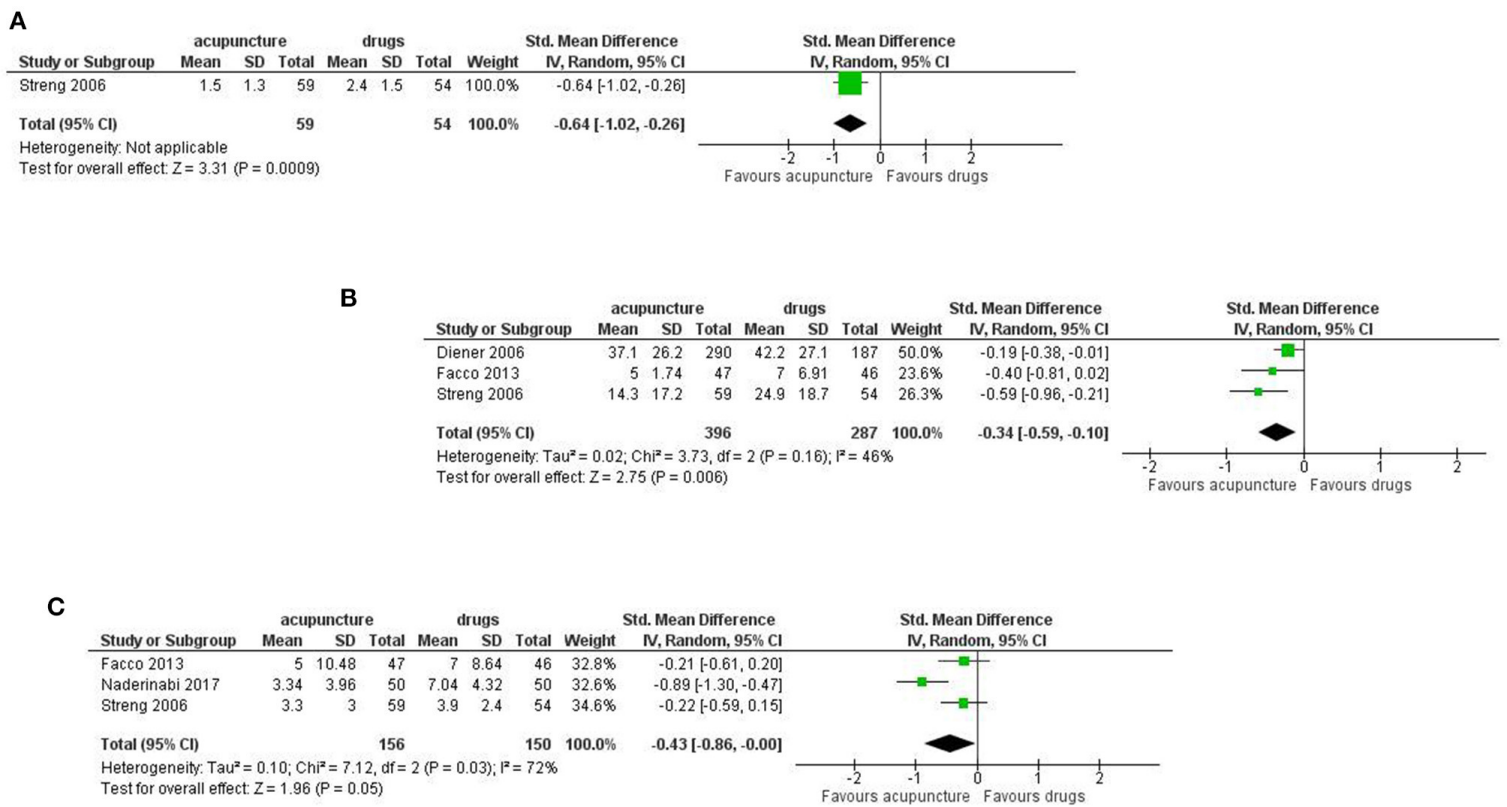
Forest plot of primary outcomes—results at longest available follow-up. **(A)** Number of days with migraine per months; **(B)** response rate to treatment; **(C)** Pain intensity.

Results in favor of acupuncture were confirmed for the number of days with migraine per month (SMD: −0.46, 95% CI −0.86 to −0.05; I^2^ = 81%, 4 studies, 766 participants, moderate quality of evidence), response rate (RR: 1.57, 95% CI 1.01 to 2.42; I^2^ = 77%, 3 studies, 730 participants, moderate quality of evidence) pain intensity (SMD: −0.37, 95% CI −0.61 to −0.14; I^2^ = 48%, 3 studies, 730 participants, moderate quality of evidence; Figures 7A–C).

**Figure 8:**
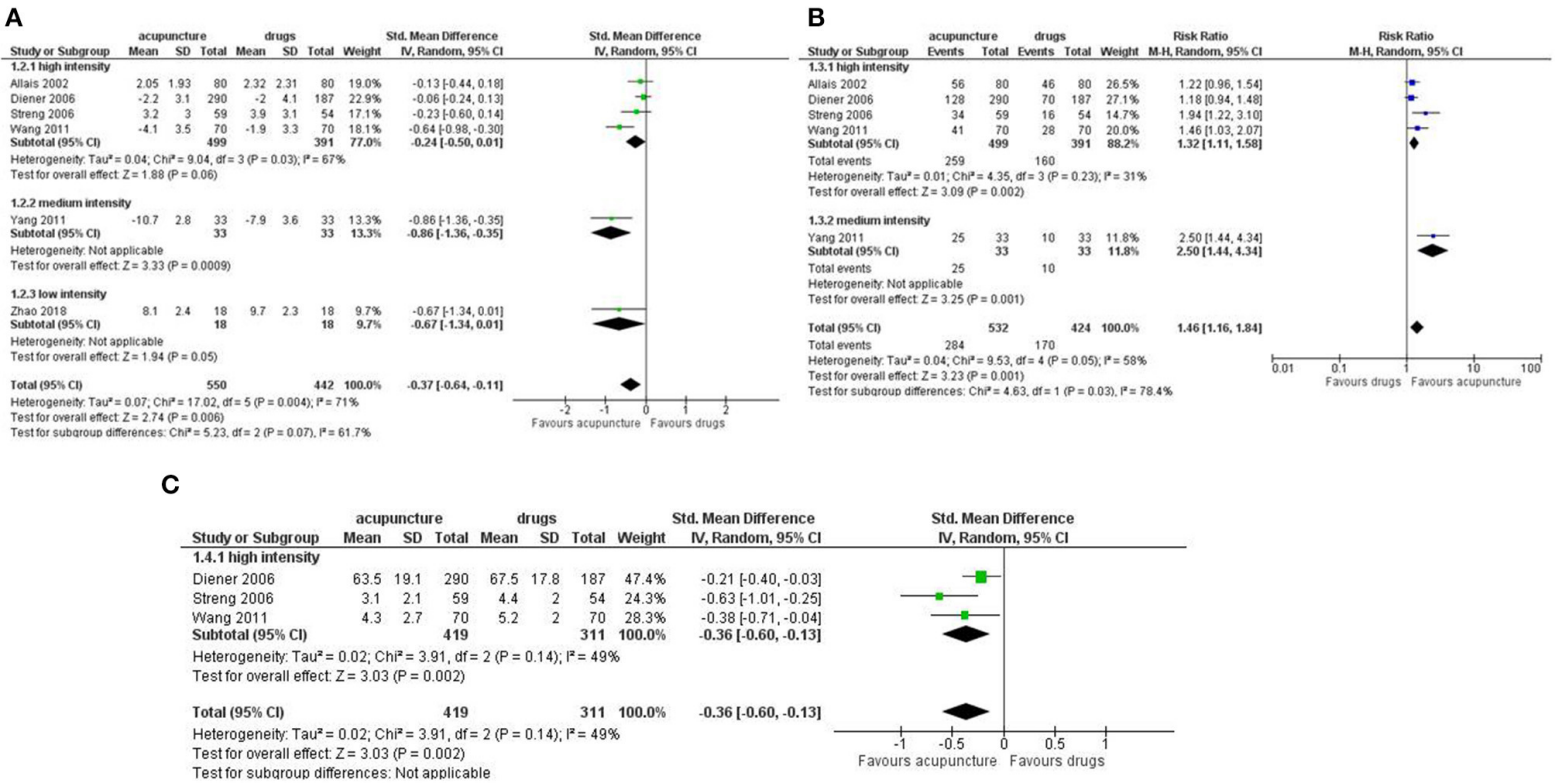
Forest plot of secondary outcomes—results at longest available follow up. **(A)** Frequency of migraine attack per month; **(B)** Disability; **(C)** Use of rescue medication.

For the secondary outcomes we found significant difference between treatments in the frequency of migraine attack per month (SMD: −0.64, 95% CI −1.02 to −0.26; 1 study, 113 participants); disability (SMD: −0.34, 95% CI −0.59 to −0.10; I^2^ = 46%, 3 studies, 683 participants); use of rescue medication (SMD: −0.43, 95% CI −0.86 to −0.00; I^2^ = 72%, 3 studies, 306 participants; Figures 8A–C).

**Figure 9:**
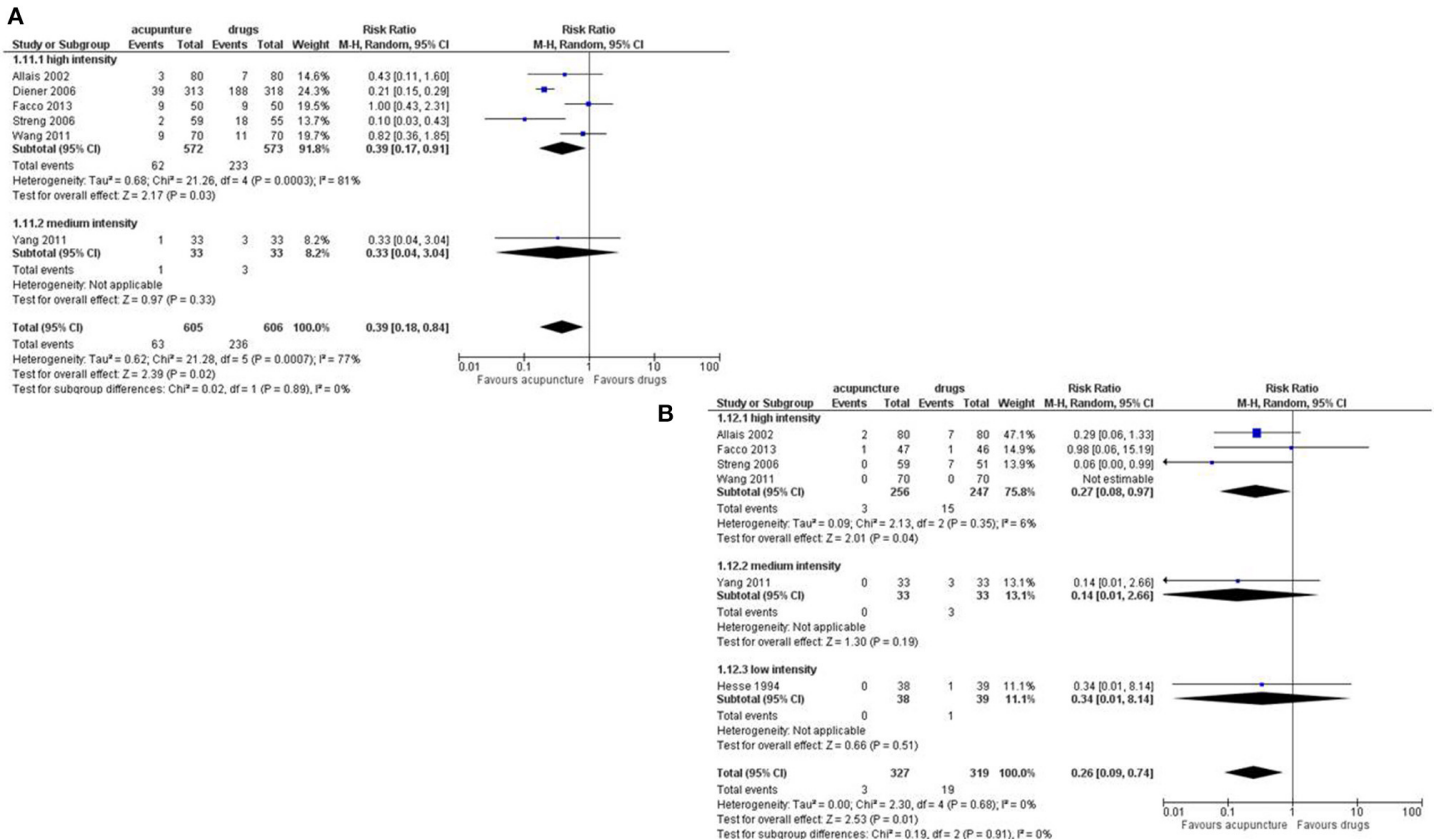
Forest plot of secondary outcomes—results at longest available follow up. **(A)** Quality of Life: mental health; **(B)** Quality of Life: physical health.

At follow up no significant difference was found for Quality of Life, both the mental health subdomain (SMD: 0.12, 95% CI −0.02 to 0.27; I^2^ = 0%, 3 studies, 730 participants) and physical health subdomains (SMD: 0.22; 95% CI −0.04 to 0.47; I^2^ = 56%, 3 studies, 730 participants; Figures 9A,B).

## Discussion

We included in our meta-analysis 9 studies, with a total of 1,484 participants that compared acupuncture with pharmacological prophylaxis for the prevention of migraine attacks in adults with chronic or episodic migraine. We found moderate quality of evidence that acupuncture is mildly more effective than any pharmacological prophylaxis in reducing the days with migraine per month, pain intensity, drop out from treatment, though the magnitude of the differences was small. We also found moderate quality of evidence that acupuncture is more effective than pharmacological prophylaxis in increasing the response rate to treatments and quality of life, but the magnitude of the differences was small. Conversely, we found moderate quality of evidence of a strong reduction in favor of acupuncture in both the dropout rate due to any and in the dropout rate due to adverse event.

Studies comparing true acupuncture vs. sham acupuncture in all its forms: superficial needling of “true” points (superficial needling of the acupoints for the treated condition), non-acupuncture' points (needling non-acupoints), “irrelevant” acupoints (needling of the acupoints not for the treated condition), “placebo needles” (devices that mimic acupuncture without skin penetration but pressing the skin) (45) have not been included, because sham acupuncture has already been proved not to be a placebo. Back in 2006, Lund et al. demonstrated that “*the light touch of the skin stimulated mechanoreceptors coupled to slow conducting unmyelinated (C) afferents resulting in activity in the insular region, but not in the somatosensory cortex. Activity in these C tactile afferents was suggested to induce a ‘limbic touch’ response resulting in emotional and hormonal reactions”* (46).

Many control procedures that were meant to be inert were likely to be activating these C tactile afferents and consequently resulted in the alleviation of the affective component of pain (46).

In the following years, numerous studies showed that sham acupuncture is just as effective as true acupuncture for the treatment of migraine (16); in addition, it may induce a wide range of peripheral, segmental, and central physiological responses to an unpredictable degree (43). Consequently, any intervention involving skin stimulation, whether it be penetration, pressure, or touch, cannot be considered an inert placebo (16, 31, 37, 38, 41, 43, 47).

Moreover, we chose to compare acupuncture against pharmacologic prophylaxis, instead of against sham acupuncture, to follow a more practical approach and provide a summary of the existing evidence that can be more useful in clinical practice as the pharmacologic prophylaxis is the most common treatment in usual care practice.

The most relevant flaws of the included studies were lack of blinding in all but two studies and the high risk of attrition bias in three studies. Overall, the certainty of evidence was judged moderate according to the GRADE approach for all the primary outcomes. A further limitation of some of the included studies is the short duration of treatment (4 weeks) and the short duration of follow up, as the effect of the medication might not be developed yet and acupuncture treatment usually last for several months. It should be noted that, in the studies with shorter follow-up the difference in treatment effect may be related to the faster mechanism of action of acupuncture compared to that of pharmacological prophylaxis. On the basis of the comparative studies currently in the literature, it was not possible to refer to very short windows of action, because these have not been considered by the authors. The exact onset of the prophylactic effect is not easily measurable and has not, however, been the subject of the studies we have considered in our review.

### Strengths and Limitations

The two most recent SRs that addressed this topic were Linde et al. (16) and Zhang et al. (18).

Linde et al. is a Cochrane systematic review, well-conducted, but not updated since 2016, and Zhang et al. did not pool data across studies.

The strength of our review relies in a comprehensive bibliographic search on several databases without time restriction and in the rigor of the methodology that followed the highest standards as recommended by Cochrane (24).

Our review has some limitations. We limited our inclusion criteria to studies published in western languages due to our inability to translate studies published in Chinese or other eastern languages. Given the widespread use of acupuncture in Eastern countries and particularly in China, we probably missed some studies that made our comparison of interest. An overview of systematic reviews recently published described 14 systematic reviews that assess the efficacy of acupuncture against sham acupuncture or pharmacologic prophylaxis; we retrieved the full text of such reviews to look for trials which we could have missed. Unfortunately, eight of these reviews were written in Chinese and included primary studies written in Chinese as well so we were unable to evaluate the included studies.

Furthermore, a limitation of some of the included studies is the short duration of treatment (4 weeks) and the short duration of follow up, as the effect of the medication might not be developed yet and acupuncture treatment usually last for several months. Finally, we were unable to visually inspect funnel plot for the presence of possible publication bias because if <10 studies are included in meta-analysis, the funnel plot in considered uninformative (24).

### Implication for Further Research

The major flaws of most retrieved studies were the lack of blinding, that poses the efficacy results at high risk of performance and detection bias. Studies that adopt a double blind, double dummy design could provide unbiased estimates of efficacy results, though, due to the nature of the intervention, a double-blind trial is hard to be conducted.

## Conclusion

Based on moderate certainty of evidence, we conclude that acupuncture is mildly more effective and much safer than medication for the prophylaxis of migraine.

## Data Availability Statement

The original contributions generated in the study are included in the article/Supplementary Material, further inquiries can be directed to the corresponding author.

## Author Contributions

SM and MC conceptualized and designed the study, screened studies from title and abstract, extracted data from included studies, assesses risk of bias, undertook data analysis, evaluated the certainty of evidence, and drafted the initial manuscript. CG, SC, and AM wrote the introduction and the discussion. All review authors contributed to writing and revising the final manuscript.

## Conflict of Interest

The authors declare that the research was conducted in the absence of any commercial or financial relationships that could be construed as a potential conflict of interest.

## Supplementary Material

Supplementary Material 1 Search strategy.
