# Supplementary material for: Acupuncture vs. Pharmacological Prophylaxis of Migraine: A Systematic Review of Randomized Controlled Trials
Source: Front Neurol. 2020 Dec 15;11:576272. doi: 10.3389/fneur.2020.576272 (PMC7773012; doi:10.3389/fneur.2020.576272)
Supplement: Supplementary Material 1 — Search strategy. [file Table_1.DOCX]

**MEDLINE 14/05/2020**

| [#32](https://www.ncbi.nlm.nih.gov/pubmed/advanced) | Search **#27 AND #28 AND #31** | [**13**](https://www.ncbi.nlm.nih.gov/pubmed/?cmd=HistorySearch&querykey=32) |
| --- | --- | --- |
| [#31](https://www.ncbi.nlm.nih.gov/pubmed/advanced) | Search **(“Propranolol"[Mesh] OR "propranolol"[Title/Abstract] OR "metopropolol"[Title/Abstract] OR "atenolol"[Title/Abstract] OR "timolol"[Title/Abstract] OR "Verapamil"[Mesh] OR "verapamil"[Title/Abstract] OR fluranizine[Title/Abstract] OR "Cinnarizine"[Mesh] OR "cinarizine"[Title/Abstract] OR "Nimodipine"[Mesh] OR "nimodipine"[Title/Abstract] OR "Topiramate"[Mesh] OR "topiramate"[Title/Abstract] OR "Valproic Acid"[Mesh] OR "valproate sodium"[Title/Abstract] OR "Lamotrigine"[Mesh] OR "lamotrigine"[Title/Abstract] OR "Amitriptyline"[Mesh] OR "amitriptyline"[Title/Abstract] OR "Duloxetine Hydrochloride"[Mesh] OR "duloxetine"[Title/Abstract] OR "Paroxetine"[Mesh] OR "paroxetine"[Title/Abstract] OR "Pizotyline"[Mesh] OR "pizotifen"[Title/Abstract] OR "Dihydroergotamine"[Mesh] OR "dihydroergotamine"[Title/Abstract] OR ciproeptadine[Title/Abstract] OR "Botulinum Toxins"[Mesh] OR "botulinum toxin"[Title/Abstract] OR (("gepant monoclonal antibody"[Title/Abstract] OR "gepant monoclonal antibodies"[Title/Abstract])** | [141233](https://www.ncbi.nlm.nih.gov/pubmed/?cmd=HistorySearch&querykey=31) |
| [#28](https://www.ncbi.nlm.nih.gov/pubmed/advanced) | Search **((("Acupuncture Therapy"[Mesh]) OR "acupuncture"[Text Word]))** | [29380](https://www.ncbi.nlm.nih.gov/pubmed/?cmd=HistorySearch&querykey=28) |
| [#27](https://www.ncbi.nlm.nih.gov/pubmed/advanced) | Search **(((("Migraine with Aura"[Mesh]) OR "Migraine without Aura"[Mesh])) OR (("Migraine Disorders"[Mesh:NoExp]) AND "Chronic Disease"[Mesh:NoExp])) OR ((("migraine with aura"[Text Word]) OR "migraine without aura"[Text Word]) OR "chronic migraine"[Text Word])** | [6742](https://www.ncbi.nlm.nih.gov/pubmed/?cmd=HistorySearch&querykey=27) |

**Embase 14/05/2020**

| **#28** | #6 AND #27 | **111** |
| --- | --- | --- |
| **#27** | #7 OR #8 OR #9 OR #10 OR #11 OR #12 OR #13 OR #14 OR #15 OR #16 OR #17 OR #18 OR #19 OR #20 OR #21 OR #22 OR #23 OR #24 OR #25 OR #26 | **392826** |
| **#26** | 'gepant monoclonal antibodies' | **0** |
| **#25** | 'botulinum toxin'/exp OR 'botulinum toxin' | **37149** |
| **#24** | 'cyproheptadine'/exp OR 'cyproheptadine' | **8387** |
| **#23** | 'dihydroergotamine'/exp OR 'dihydroergotamine' | **5951** |
| **#22** | 'pizotifen'/exp OR 'pizotifen' | **1976** |
| **#21** | 'paroxetine'/exp OR 'paroxetine' | **27688** |
| **#20** | 'duloxetine'/exp OR 'duloxetine' | **10767** |
| **#19** | 'amitriptyline'/exp OR 'amitriptyline' | **39774** |
| **#18** | 'lamotrigine'/exp OR 'lamotrigine' | **24688** |
| **#17** | 'valproic acid'/exp OR 'valproic acid' | **63075** |
| **#16** | 'valproate semisodium'/exp OR 'valproate semisodium' | **5443** |
| **#15** | 'topiramate'/exp OR 'topiramate' | **22051** |
| **#14** | 'nimodipine'/exp OR 'nimodipine' | **10568** |
| **#13** | 'cinnarizine'/exp OR 'cinnarizine' | **2636** |
| **#12** | 'flunarizine'/exp OR 'flunarizine' | **5159** |
| **#11** | 'verapamil'/exp OR 'verapamil' | **58048** |
| **#10** | 'timolol'/exp OR 'timolol' | **14753** |
| **#9** | 'atenolol'/exp OR 'atenolol' | **31565** |
| **#8** | 'metoprolol'/exp OR 'metoprolol' | **36104** |
| **#7** | 'propranolol'/exp OR 'propranolol' | **100844** |
| **#6** | #4 AND #5 | **216** |
| **#5** | 'acupuncture'/exp OR 'acupuncture' OR auriculotherapy | **51605** |
| **#4** | #1 OR #2 OR #3 | **12585** |
| **#3** | 'migraine without aura'/exp OR 'migraine without aura' | **4752** |
| **#2** | 'migraine with aura'/exp OR 'migraine with aura' | **5648** |
| **#1** | 'transformed migraine'/exp OR 'transformed migraine' OR 'chronic migraine' | **5518** |

**Cochrane Library 14/05/2020**

**#1 MeSH descriptor: [Migraine with Aura] explode all trees 109**

**#2 MeSH descriptor: [Migraine without Aura] explode all trees 124**

**#3 MeSH descriptor: [Migraine Disorders] explode all trees 2416**

**#4 MeSH descriptor: [Chronic Disease] explode all trees 12736**

**#5 #3 AND #4 86**

**#6 "migraine with aura" OR "migraine without aura" OR "chronic migraine" 1483**

**#7 #1 OR #2 OR #5 OR #6 1499**

**#8 MeSH descriptor: [Acupuncture] explode all trees 141**

**#9 acupuncture 15585**

**#10 #8 OR #9 15585**

**#11 #7 AND #10 79**

**#12 "propranolol" OR "metopropolol" OR "atenolol" OR "timolol" OR "verapamil" OR fluranizine OR "cinarizine" OR "nimodipine" OR "topiramate" OR "Valproic Acid" OR "valproate sodium" OR "lamotrigine" OR "amitriptyline" OR "duloxetine" OR "paroxetine" OR "pizotifen" OR "dihydroergotamine" OR ciproeptadine OR "botulinum toxin" OR "gepant monoclonal antibody" OR "gepant monoclonal antibodies" 26524**

**#13 #11 AND #12 16**
